# Crimean-Congo Hemorrhagic Fever Virus in Ticks, Southwestern Europe, 2010

**DOI:** 10.3201/eid1801.111040

**Published:** 2012-01

**Authors:** Agustín Estrada-Peña, Ana M. Palomar, Paula Santibáñez, Nely Sánchez, Miguel A. Habela, Aránzazu Portillo, Lourdes Romero, José A. Oteo

**Affiliations:** University of Zaragoza, Zaragoza, Spain (A. Estrada-Peña, N. Sánchez);; Hospital San Pedro–CIBIR, La Rioja, Spain (A.M. Palomar, P. Santibáñez, A. Portillo, L. Romero, J.A. Oteo);; University of Extremadura, Cáceres. Spain (M.A. Habela)

**Keywords:** Crimean-Congo hemorrhagic fever virus, CCHFV, hemorrhagic fever, virus, ticks, *Hyalomma* spp., southwestern Europe, Mediterranean Basin

**To the Editor:** Crimean-Congo hemorrhagic fever virus (CCHFV; family *Bunyaviridae*, genus *Nairovirus*) causes outbreaks of severe hemorrhagic fever in humans, with case-fatality rates <30% (*1**,**2*). The disease was initially recognized by Russian scientists in the 1940s (*3*), and the virus was first isolated in the Democratic Republic of Congo some years later (*4*). CCHFV is reported throughout broad regions of Africa, Europe, the Middle East, and Asia. Reports linking transmission of the virus with an infected vector have involved ticks of the genus *Hyalomma* (*5*). It appears that maintenance of active foci of CCHFV in the field is dependent on *Hyalomma* spp., even within periods of silent activity. Several vertebrates are involved in the natural transmission cycle (*6*). Transmission of CCHFV to humans occurs through tick bites, direct contact with blood or tissues of infected animals, person-to-person spread, or by nosocomial infection (*1*).

In southeastern Europe, the Balkans are the known western limit for CCHFV (*7*). This finding is of special interest because *Hyalomma marginatum*, the main tick vector in the western Paleartic (an ecozone that includes temperate and cold areas of Eurasia and North Africa and several archipelagos and islands in the Atlantic and Pacific Oceans), is common throughout the Mediterranean Basin (*7*), where clinical cases of the disease or the virus have not been reported. Unsupported claims of the effects of climate on virus distribution have been reported but never empirically demonstrated (*8*).

We report the detection of CCHFV in ticks collected in southwestern Europe. A total of 117 semi-engorged adult *H. lusitanicum* ticks were collected from 28 adult red deer (*Cervus elaphus*) in November 2010, at a site (39.63°N, 7.33°W) in Cáceres, Spain. Live ticks were transported to the special pathogens laboratory at Hospital San Pedro–CIBIR in Logroño (northern Spain), classified, and frozen at −80°C. For RNA extraction, specimens were washed in 70% ethanol and then in Milli-Q water (Milli-Q Advantage water system; Millipore Ibérica, S.A., Madrid, Spain) that had been autoclaved. Each tick was cut lengthwise; half was used for additional processing and the remainder was stored. Before use, each half was crushed in sterile conditions. RNA was individually extracted by using the RNeasy Mini Kit (QIAGEN, Hilden, Germany) according to the manufacturer’s instructions and frozen at −80°C.

The RNA was distributed in 12 pools and retrotranscribed by using the Omniscript RT kit (QIAGEN) according to the manufacturer’s instructions and then frozen at −20°C. Nested PCRs were performed by using specific primers for the small segment of CCHFV as described (*9*). Negative controls (with template DNA but without primers and with primers and containing water instead of template DNA) were included in all assays.

For the second round of PCRs, 2 of 12 pools showed amplicons of the expected size (211 bp). Only 1 amplicon could be sequenced. MEGA5 (www.megasoftware.net) was used to compare the sequence with representative small segment sequences of CCHFV available in GenBank (Figure). (Aligned sequences are available from the authors.) Pools of cDNA were submitted to the Spanish National Center of Microbiology (Madrid), where results were confirmed. The CCHFV sequence we report showed 98% genetic similarity (204/209 bp) with sequences recorded for CCHFV in Mauritania and Senegal, on the western coast of Africa.

**Figure:**
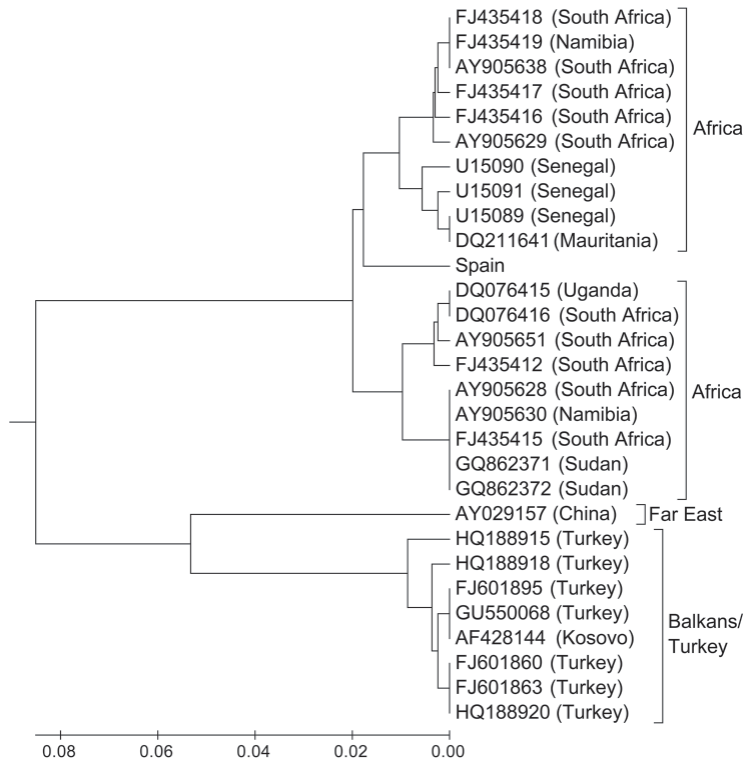
Evolutionary relationships of Crimean-Congo hemorrhagic fever virus strains from Spain and other representative sites. Evolutionary history was inferred by using the unweighted pair group method with arithmetic mean. The optimal tree is shown (sum of branch length, 0.36861921). The tree is drawn to scale, with branch lengths in the same units as those of the evolutionary distances used to infer the phylogenetic tree. Evolutionary distances were computed by using the maximum composite likelihood method and are in the units of the no. of base substitutions per site. Analysis involved 29-nt sequences. The first, second, third, and noncoding codon positions were included. All positions containing gaps and missing data were eliminated. Evolutionary analyses were conducted by using MEGA5 (www.megasoftware.net).

This finding suggests the circulation of CCHFV in southwestern Europe. The close affinity of the strain from Spain with strains circulating in western Africa and the lack of similarity with isolates from eastern Europe suggest the introduction of this virus from nearby countries of northern Africa. Migratory movements of birds could explain the presence of the virus in southwestern Europe because birds are common hosts of immature *H. marginatum*, which was reportedly introduced into Europe through annual migratory flights along the western coast of Africa (*10*). Because of the lack of genetic similarities among virus strains, trade movements of domestic or wild ungulates from eastern Europe do not support our finding.

We cannot state whether this virus was circulating previously or if other strains are present in the area because CCHFV detection in the western Mediterranean region has not been previously addressed. *H. lusitanicum* ticks exist as relatively isolated populations in a narrow strip from Sicily to Portugal. The Mediterranean rabbit and ungulates, the main hosts for immature and adult *H. lusitanicum* ticks, respectively, are residents of the collection area; however, the movement of these animals through trade has not occurred for several years. Thus, *H. lusitanicum* ticks could not serve as a spreading vector in the western Mediterranean region. The CCHFV strain from southwestern Europe has been found in ticks restricted to hosts that cannot spread long distances. Therefore, although it would be unlikely, given the strain’s similarity with CCHFV isolates from Senegal and Mauritania, we should not exclude the possibility of an ancient existence for this strain. Additional data collected in the Mediterranean Basin are necessary to establish the actual range of CCHFV.
